# Venous thromboembolism prophylaxis regimen for patients undergoing deep inferior epigastric perforator flap breast reconstruction^✰^

**DOI:** 10.1016/j.jpra.2026.02.024

**Published:** 2026-02-27

**Authors:** Taylor G. Kreul, Jacob C. Koster, Arad Arasteh, Ricardo Gomez, Haley A. Kenner, Alma Jukic, Santana S. Solomon, Janelle Rodriguez, Irene Ma, Michel Saint-Cyr

**Affiliations:** a University of Arizona College of Medicine - Phoenix, 475 North 5th Street Phoenix, AZ, USA; b Department of Surgery Mayo Clinic School of Graduate Medical Education, Mayo Clinic College of Medicine and Science, 200 First Street Southwest Rochester, Minnesota, USA; c Department of General Surgery, Banner - University Medicine Center Phoenix, 1111 East McDowell Road, Phoenix, AZ, USA; d Banner Gateway Medical Center, Banner MD Anderson Cancer Center, 2946 East Banner Gateway Drive, Gilbert, AZ, USA; e Division of Plastic and Reconstructive Surgery, Mayo Clinic Arizona, 5777 East Mayo Boulevard, Phoenix, AZ, USA

**Keywords:** Breast reconstruction, DIEP flap, Prophylaxis protocol, Venous thromboembolism

## Abstract

**Background:**

Patients undergoing deep inferior epigastric perforator (DIEP) flap breast reconstruction are at increased risk for venous thromboembolism (VTE) due to prolonged operative times, cancer-associated hypercoagulability, and postoperative immobility. Despite this risk, consensus is limited regarding standardized prophylaxis regimens for this population. We assessed VTE incidence in patients undergoing DIEP flap breast reconstruction after implementing a standardized, multimodal VTE prophylaxis regimen.

**Methods:**

We retrospectively reviewed patients who underwent DIEP flap breast reconstruction at our institution between October 2021 and July 2024. Patients were subjected to a standardized VTE prophylaxis regimen including preoperative education and subcutaneous heparin injection, intraoperative efficiency techniques, and a postoperative protocol consisting of early ambulation, sequential compression devices, compressive garments, and a 2-week course of enoxaparin sodium. Patient characteristics, operative details, and VTE outcomes were analyzed.

**Results:**

In a cohort of 110 patients, 187 DIEP flap reconstruction procedures were performed by one surgeon. Mean patient age was 49.3 years, and mean body mass index was 29.7. The VTE rate directly attributable to DIEP flap reconstruction was 0%. One VTE event occurred—an upper extremity deep vein thrombosis (DVT) 119 days postoperatively that was associated with intravenous access during a revision procedure. No lower-extremity DVTs or pulmonary embolisms were reported.

**Conclusions:**

Implementation of a standardized, comprehensive VTE prophylaxis regimen encompassing preoperative, intraoperative, and postoperative measures can result in a remarkably low VTE rate in patients undergoing DIEP flap breast reconstruction. This safe, effective regimen may eliminate the need for risk-based pharmacologic stratification tools such as the Caprini model.

## Introduction

Postoperative venous thromboembolism (VTE) is a rare but serious surgical complication that can result in morbidity and mortality.^1^^,^^2^ Breast cancer survivors who undergo reconstruction often have various risk factors for VTE, such as prolonged operative times, underlying malignancy, use of immunotherapies, and limited mobility after the procedure.^2^, ^3^, ^4^ The incidence of VTE in patients undergoing breast reconstruction is 3.2%, although this number ranges from 0.8% to 31.4%, depending on the study.^5^, ^6^, ^7^ A recent systematic review of patients undergoing microsurgical breast reconstruction showed a 1.01% overall rate of deep vein thrombosis (DVT) and a 0.55% rate of pulmonary embolism (PE).^8^ Moreover, autologous breast reconstruction, such as deep inferior epigastric perforator (DIEP) flap reconstruction, confers higher odds of VTE than implant reconstruction or mastectomy alone, mostly due to prolonged operative times.^4^ Therefore, VTE risk must be accurately stratified and appropriately managed in the postoperative setting.

The Caprini risk assessment model (RAM) is a validated tool used to assess the risk of VTE in patients undergoing surgery. Those undergoing major reconstructive surgery, such as DIEP flap reconstruction, typically have a Caprini score of 3 or higher, which is associated with a moderate-to-high risk of VTE. Prior studies using the Caprini RAM have consistently shown that patients undergoing DIEP flap breast reconstruction usually fall into the high-risk category.^5^^,^^9^

Despite its widespread use, the utility of the Caprini RAM for patients undergoing breast reconstruction is often debated due to the exclusion of potential risk factors such as certain types of anesthesia, prior radiotherapy and chemotherapy, and *BRCA* sequence variation. Due to disagreement about the usefulness of various VTE risk assessment measures, a standardized approach for VTE prophylaxis is needed for patients undergoing DIEP flap breast reconstruction.

Controlling for risk factors of VTE throughout the process of patient care may be as important as pharmacologic prophylaxis alone. Although numerous studies have examined the role of pharmacologic agents in VTE prophylaxis, few have examined a holistic approach to VTE prevention.^3^^,^^10^, ^11^, ^12^ We hypothesized that key changes in surgical techniques combined with the standardization of patient education, pharmacologic intervention, and shortened length of hospital stay would decrease the VTE rate in our patient population.

## Methods

### Study design

A retrospective chart review was conducted for all patients at our institution who underwent DIEP flap breast reconstruction after nipple-sparing mastectomy due to breast cancer or a high-risk genetic variant for breast cancer at Banner MD Anderson Cancer Center in Gilbert, Arizona between October 1, 2021, and July 31, 2024. Patients who underwent bilateral or unilateral DIEP flap breast reconstruction were included. The primary surgeon (M. S.-C.) was the same for all cases. Pediatric patients (ie, <18 years of age) were excluded. All patients were subjected to a standardized VTE prophylaxis regimen that was developed in accordance with the Enhanced Recovery After Surgery (ERAS) protocol to optimize postoperative recovery and enhance patient outcomes.^13^ The regimen (Box 1) was designed to universally minimize VTE risk, reduce health care team burden, and prioritize patient well-being and autonomy. This regimen was used for all patients undergoing DIEP flap breast reconstruction, regardless of their individual risk factors for VTE.

**Box 1:** Venous thromboembolism prophylaxis regimen.

**Preoperative measures** Comprehensive preoperative education that informs patients about their postoperative course to enhance their understanding and preparation for early discharge. Subcutaneous heparin injection received in the preoperative care unit. **Intraoperative measures** Shorter fascial incisions and pedicle lengths used to minimize the length of operative time and reduce the pressure on the femoral and iliac veins. Pain control provided via transversus abdominis plane blocks and incisional infiltration analgesia. **Postoperative measures** Patient discharge on postoperative day 1. Two-week course of enoxaparin sodium [Lovenox, Sanofi Winthrop Industrie, France], injected once daily (40 mg) for 14 days. Use of lower-extremity sequential compression devices and compressive lower-extremity garments. Early postoperative mobilization, with in-hospital physical therapy beginning the day after surgery at 8:00 am to promote early ambulation. Prioritization of patient education and safety regarding early mobility.

### Data collection and analysis

Patient records were reviewed, and data were collected and analyzed. Preoperative data included patient demographic information (age), body mass index (BMI) calculated as weight in kilograms divided by height in meters squared, and comorbid conditions. Operative data included the type of procedure, laterality, and immediate vs delayed reconstruction. Postoperative data included postoperative hospital length of stay and major and minor complications. Major complications included VTE (PE and DVT), flap loss, infection requiring reoperation, venous congestion requiring reoperation, sepsis, and infection requiring readmission. Minor complications included seroma, hematoma, cellulitis, fat necrosis, dehiscence, skin necrosis, and delayed wound healing. VTE risk was also calculated retrospectively for all patients by using the Caprini RAM 2005.^14^ Patients with scores of 3 to 4 were categorized as high risk, and those with scores of 5 or more were categorized as having the highest risk. Study data were collected and managed by using REDCap (Research Electronic Data Capture) hosted at University of Arizona.^15^^,^^16^ REDCap is a secure, web-based software platform designed to support data capture for research studies. Data were analyzed by using Microsoft Excel (Microsoft Corporation).

## Results

**Figure 1:**
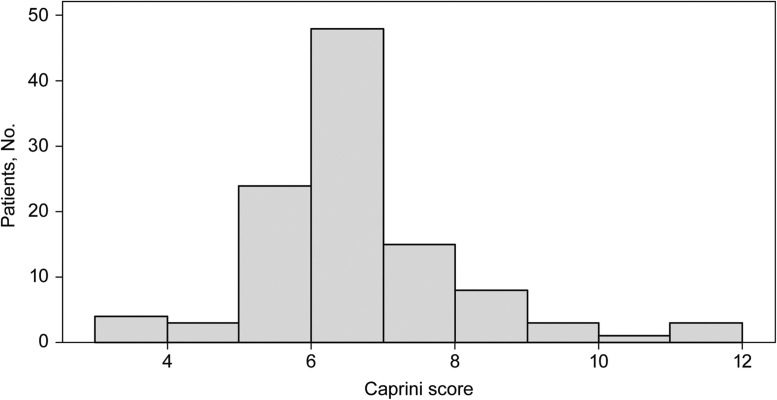
Distribution of Caprini Scores. The mean Caprini score was 6.2, with a range of 3–11 and a mode of 6. Patients with scores of 3 to 4 are categorized as high risk, with a 20% to 40% incidence of deep vein thrombosis.^14^ Patients with scores of 5 or more are categorized as the highest risk, with a 40% to 80% incidence of deep vein thrombosis and a mortality rate of 1% to 5%.^14^

A total of 187 DIEP flap breast reconstruction procedures were performed for 110 patients. Mean patient age at the time of DIEP flap reconstruction was 49.3 years old, and mean BMI was 29.7 (Table 1). Mean Caprini score was 6.2, with all patients falling within the categories of high and highest risk (Figure 1).^14^ Comorbid conditions are summarized in Table 1.

**Table 1:** Patient characteristic^a^ (*N* = 110).

Variable	Value
Age, mean (range), y	49.3 (29–67)
BMI^b^, mean (range)	29.7 (21.1–45.4)
Comorbid condition	
Diabetes	2 (1.8)
Hypertension	14 (12.7)
Coagulopathy	1 (0.9)
Caprini score, mean (range)	6.2 (3–11)

Abbreviation: BMI, body mass index.

^a^Data are expressed as No (percentage) unless otherwise indicated.

^b^Calculated as weight in kilograms divided by height in meters squared.

Immediate reconstruction was performed in seven cases (3.7%), and delayed reconstruction was performed in 180 cases (96.3%) (Table 2). Of the total flap reconstructions performed, 160 (85.6%) were for bilateral reconstruction, whereas 27 (14.4%) were for unilateral reconstruction. Although the standardized postoperative pathway targeted discharge on postoperative day 1, actual discharge timing was individualized based on clinical recovery, comorbidities, and social factors, resulting in a mean hospital stay of 2.5 days. The overall flap viability rate was 99.5%, with the loss of only one DIEP flap due to intrinsic venous congestion and flap scarring. Only one case of VTE was reported, which was an upper-extremity DVT that occurred 119 days after a revision procedure. No cases of PE were reported. Thus, the overall incidence of VTE was 0.5%, with a DVT rate of 0.5% and a PE rate of 0% (Table 2).

**Table 2:** Operative data and postoperative outcomes^a^ (*N* = 187).

Variable	Value
DIEP flaps	
Unilateral	27 (14.4)
Bilateral	160 (85.6)
Reconstruction	
Immediate	7 (3.7)
Delayed	180 (96.3)
Length of surgery, mean, min	456.0
Postoperative hospital length of stay, mean, d	2.5
Major complications	
VTE	1 (0.5)
DVT	1 (0.5)
PE	0
Unplanned return to the OR	6 (3.2)
Flap loss	1 (0.5)
Minor complications	
Wound dehiscence	14 (7.5)
Seroma	9 (4.8)
Fat necrosis	5 (2.7)
Skin necrosis	5 (2.7)
Delayed wound healing	4 (2.2)
Cellulitis	3 (1.6)
Hematoma	1 (0.5)
Infection requiring IV antibiotics	3 (1.6)

Abbreviations: DIEP, deep inferior epigastric perforator; DVT, deep vein thrombosis; IV, intravenous; OR, operating room; PE, pulmonary embolism; VTE, venous thromboembolism.

a Data are expressed as No (percentage) unless otherwise indicated.

### Case presentation

As mentioned above, only one case of VTE occurred in this cohort. Specifically, this was a case of upper-extremity DVT that was related to an intravenous line. The DVT did not occur immediately after the DIEP flap breast reconstruction procedure but 1 day after a bilateral DIEP revision surgery that was performed 119 days after the initial surgery. The patient was 44 years old at the time of the surgery and had a BMI of 24.2. The patient’s preoperative Caprini score was 8. She tolerated the initial DIEP flap breast reconstruction well with no complications. The patient reported a history of DVT in the same extremity, which was also provoked by an intravenous line. She reported stopping her prescribed apixaban before having the revision surgery. The patient’s care was managed while she was in the emergency department, and she recovered without incident. Test results for coagulation disorders were negative.

## Discussion

The results of implementing a standardized, comprehensive prophylaxis regimen showed a low VTE incidence of 0.5%, with only one case of upper-extremity DVT in a patient with a history of provoked DVT. According to the American College of Physicians, the risk of VTE is highest between weeks five and six after nonorthopedic surgery, but the risk remains increased for at least 12 weeks.^17^ Because the single instance of DVT in our cohort occurred nearly 17 weeks after the initial DIEP flap breast reconstruction, this case of DVT was probably unrelated to the initial DIEP procedure.

The low VTE incidence in our cohort contrasts with the wide range of VTE rates reported in previous studies of patients who underwent breast reconstruction (0.8−31.4%).^5^, ^6^, ^7^ A significant contributor to this disparity could be differences in VTE prophylaxis regimens, patient populations, and methods of outcome assessment. Importantly, our cohort consisted of patients receiving a uniform VTE prophylaxis regimen, regardless of their individual risk factors. The application of a standardized approach to VTE prevention probably contributed to the low VTE rate observed in our study. In a similar study, a standardized VTE prophylaxis protocol was used that included preoperative sequential compression devices (SCDs) and aspirin (325 mg per rectum), early postoperative ambulation, a subcutaneous injection of low-molecular-weight heparin on postoperative day 1, enoxaparin sodium (40 mg) and daily SCDs until discharge on postoperative day 3, and oral aspirin (81 mg) daily for 30 days postoperatively.^18^ In that study, five cases of PE and one case of DVT were reported, with an overall VTE prevalence of 1.1%. The lower VTE incidence observed in our study could be due to differences in preoperative, intraoperative, and postoperative details between protocols.

Several studies have explored the usefulness of the Caprini RAM for VTE risk stratification in patients undergoing breast reconstruction.^5^^,^^9^^,^^10^^,^^19^^,^^20^ Some studies have raised questions regarding the model’s effectiveness in accurately predicting VTE risk in patients undergoing DIEP flap breast reconstruction. Previously, the method of breast reconstruction (eg, transverse rectus abdominis myocutaneous flap, latissimus dorsi flap, or implant based) was shown to be a better predictor of VTE risk than the Davison risk score or the 2005 Caprini score, which were similar across all reconstruction groups.^9^ Notably, low VTE rates were reported for patients who underwent DIEP flap breast reconstruction, despite that Caprini RAM guidelines based on patient risk were not followed.^10^ In another cohort of 192 patients undergoing DIEP flap breast reconstruction, 92.1% were classified by the Caprini RAM as having a high risk for VTE and were recommended to receive prolonged VTE prophylaxis.^5^ Although some surgeons continue to avoid pharmacologic VTE prophylaxis due to concerns of hematoma formation and flap failure, data suggest that the use of enoxaparin sodium within 4 weeks postoperatively does not increase the risk of hematoma formation and is effective for reducing the risk of VTE.^12^^,^^21^

Although RAMs such as the Caprini RAM may be useful in some surgical populations, our study suggests that VTE prophylaxis performed in this patient population according to a comprehensive and standardized protocol may be effective without RAMs. Notably, our cohort included patients with varying comorbid conditions, operative characteristics, and procedural complexities. However, all were treated with the same standardized prophylaxis regimen, which led to a remarkably low VTE rate.

Our study highlights the critical role of preoperative education in preparing patients undergoing DIEP flap breast reconstruction for recovery, encouraging active participation in their postoperative care, and decreasing the risk of complications such as VTE.^22^ Comprehensive information provided about the surgical procedure, its associated risks, postoperative expectations, and the importance of early ambulation during recovery empowers patients to manage their postoperative course more effectively. Patients who are educated on how mobilization enhances circulation and reduces the risks associated with prolonged immobility may be more likely to adhere to recommendations aimed at preventing complications such as VTE. Furthermore, this education may alleviate patient anxiety, potentially leading to better recovery outcomes.

Early postoperative ambulation is a key factor in minimizing VTE risk. Encouraging patients to begin physical therapy and light activity as early as the day after surgery can reduce the time spent immobile. Early ambulation promotes venous return, enhances circulation, and decreases the likelihood of blood stasis, which is a prerequisite for clot formation. Early intervention with physical therapy can also help in preventing other postoperative complications such as muscle atrophy, joint stiffness, and DVT.^23^^,^^24^ This proactive approach aligns with the concept of reducing risks by preventing immobility-induced complications before they arise.

Our holistic approach in which we combine both pharmacologic and nonpharmacologic interventions represents a comprehensive strategy for minimizing VTE risk in patients undergoing DIEP flap breast reconstruction in accordance with ERAS protocols.^13^ By addressing both the physiologic and behavioral aspects of VTE prevention, our multifaceted approach is not only effective but also potentially superior to pharmacologic prophylaxis alone. This strategy may be particularly valuable for patients undergoing high-risk surgical procedures in which the combination of extended operative times, postoperative immobility, and underlying comorbid conditions contributes to an increased risk of VTE. Notably, following completion of the study period, the prophylaxis regimen was transitioned to apixaban (2.5 mg orally twice daily) in an effort to improve patient adherence. During the study period, postoperative pharmacologic prophylaxis consisted of enoxaparin sodium according to institutional protocol. Therefore, outcomes reported in the present analysis reflect the enoxaparin-based protocol and were not influenced by the subsequent change in prophylactic agent. Ongoing work is evaluating the safety and efficacy of apixaban in patients undergoing DIEP flap breast reconstruction, including its long-term effects in higher-risk populations.

## Conclusion

This study suggests that a standardized VTE prophylaxis regimen encompassing comprehensive preoperative, intraoperative, and postoperative measures can effectively reduce the risk of VTE in patients undergoing DIEP flap breast reconstruction. Despite that a formal risk stratification model was not used for these patients, our findings indicate that a comprehensive approach to VTE prevention can lower VTE incidence. This study challenges the usefulness of the Caprini RAM for patients undergoing DIEP flap breast reconstruction and supports the idea that a uniform, standardized prophylaxis protocol may be equally as effective, if not more effective, in minimizing VTE risk.

## Declaration of AI and AI-assisted technologies in the writing process

During the preparation of this work the author(s) used ChatGPT in order to facilitate grammatical editing. After using this tool/service, the author(s) reviewed and edited the content as needed and take(s) full responsibility for the content of the publication.

## Funding sources

None.

## Ethics statement

This study was conducted in accordance with the ethical standards of the institutional research committee and with the 1964 Helsinki Declaration and its later amendments. Institutional Review Board approval was obtained prior to data collection. Due to the retrospective nature of the study and the use of de-identified data, informed consent was waived by the Institutional Review Board.

## Declaration of competing interest

None of the authors has a financial interest in any of the products, devices, or drugs mentioned in this manuscript. Michel Saint-Cyr MD, MHA, MBA, FRCSC, FACS is the Chief Medical Officer of Sage Spectra. The authors have no other disclosures to report.
